# Prognostic effect of matrix metalloproteinase-9 in patients with resected Non small cell lung cancer

**DOI:** 10.1186/s13019-015-0248-3

**Published:** 2015-03-27

**Authors:** Chang Young Lee, Hyo Sup Shim, Seokkee Lee, Jin Gu Lee, Dae Joon Kim, Kyung Young Chung

**Affiliations:** 1 Department of Thoracic and Cardiovascular Surgery, Yonsei University College of Medicine, 250 Seongsanno Seodaemun-Gu, CPO box 8044, Seoul, South Korea; 2 Department of Pathology, Yonsei University College of Medicine, Seoul, South Korea; 3 Department of Thoracic and Cardiovascular Surgery, Armed Forces Capital Hospital, Seongnam, South Korea

**Keywords:** Matrix metalloproteinase-9 (MMP-9), Immunohistochemistry, Non-small cell lung cancer (NSCLC), Lung adenocarcinoma, Prognostic factor

## Abstract

**Background:**

The role of tumor matrix metalloproteinase-9 (MMP-9) expression in non-small cell lung cancer (NSCLC) remains controversial. In this study, we investigated the prognostic value of tumor MMP-9 expression and other clinicopathologic factors in patients with completely resected NSCLC.

**Methods:**

This retrospective study included patients who underwent complete resection of pathological stage I–IIIA NSCLC at Severance Hospital, Seoul, Korea, between 1998 and 2009. Tumor samples of 417 patients were stained by immunohistochemistry, and the expression of MMP-9 in tumor cells was evaluated, using the median immunohistochemical score of 10 (range, 0-300) as the cut-off.

**Results:**

Tumor MMP-9 expression was observed in 161 (38.6%) of 417 patients. Log-rank analysis showed a significant association of tumor MMP-9 expression with shortened disease-free survival (*p* = 0.01) but not with overall survival (*p* = 0.109). Multivariate analysis demonstrated that tumor MMP-9 expression was not an independent prognostic factor of recurrence (*p* = 0.142) or survival (*p* = 0.807). However, among patients with adenocarcinoma, tumor MMP-9 expression was associated with relapse (*p* = 0.003) and poor survival (*p* = 0.033). Furthermore, tumor MMP-9 expression was an independent prognostic indicator of relapse in patients with adenocarcinoma (*p* = 0.035).

**Conclusions:**

Among patients with NSCLC, tumor MMP-9 expression was associated with poor outcomes in those with adenocarcinoma, but not in those with squamous cell carcinoma. In addition, MMP-9 expression was identified as an independent predictor of relapse of completely resected lung adenocarcinoma.

## Background

Lung cancer is the leading cause of cancer death in the United States, and the 5-year survival for non-small cell lung cancer (NSCLC) of all stages is only approximately 15% [1,2]. Surgical resection is typically performed for early-stage NSCLC, however even among patients whose tumors are successfully resected, the 5-year survival rate is only 50–60%, and for certain patients, recurrence occurs within a few years following resection. In addition, NSCLC patients with the same stage may show different patterns of disease progression [3-5]. Therefore, it is important to identify molecular prognostic markers for NSCLC that may guide the use of adjuvant therapy after surgical resection.

Numerous studies have demonstrated that the expression of matrix metalloproteinases (MMPs) is associated with lung cancer prognosis [6-8]. MMPs are associated with degradation of the extracellular matrix and are thought to play important roles in tumor invasion and metastasis [9]. Among the many MMPs, MMP-9 (gelatinase-B), a 92-kDa gelatinase that can catalyze type IV collagen in the basal membrane, is considered a key enzyme. MMP-9 has been reported to facilitate tumor growth, invasion, and angiogenesis [10,11].

Recently, many studies have used immunohistochemical (IHC) analysis to investigate MMP-9 expression in resected tumors. These have demonstrated a correlation between MMP-9 expression and prognosis [12-15]. However, the clinical efficacy of tumor MMP-9 expression as a prognostic marker in patients with operable NSCLC remains controversial [16,17]. Moreover, there is disagreement on how best to define positive IHC staining for MMP-9.

This study was designed to investigate the expression of tumor MMP-9 in operable NSCLC and to analyze the relationship between tumor MMP-9 expression and prognosis. Furthermore, this study assessed the impact of tumor MMP-9 expression on the prognosis and outcome of patients with operable NSCLC.

## Methods

### Patients

This was a retrospective study of 473 patients with stage I–IIIA NSCLC who underwent radical resection of primary lung cancer at Severance Hospital between 1998 and 2009. This study was approved by the Institutional Review Board of the Yonsei University College of Medicine. The IRB waived the requirement of individual patient consent because the analysis was retrospective in nature. Patients were excluded according to the following criteria: (1) radiotherapy or chemotherapy prior to surgery, (2) tumor tissue not available, (3) pathological stage IIIB or stage IV disease, (4) complete resection not achieved (not R0), and (5) postoperative survival <60 days.

In total, 417 patients met the selection criteria and were included in the analysis. Preoperative evaluations included routine chest radiography, bronchoscopy, computed tomography of the chest, abdominal sonography, and a bone scan or 18 F-fluorodeoxyglucose positron emission tomography (^18^FDG-PET). Post-operatively, follow-up was achieved through regular clinic visits until the patient’s death. Patients were examined by chest computed tomography at 3-month intervals for 2 years and at 6-month intervals thereafter. Furthermore, abdominal sonography or ^18^FDG-PET or bone scans was performed at 6-month intervals for 5 years and at 1-year intervals thereafter, and since 2007 ^18^FDG-PET has been used instead of abdominal sonography.

Paraffin-embedded tumor specimens were used to create tissue microarray blocks with 2-mm diameter cores for IHC staining. Two tissue cores were obtained from each patient. Pathologic staging was classified according to the 7^th^ edition of the Union for International Cancer Control tumor-node-metastasis classification of lung cancer.

### Immunohistochemical staining

Formalin-fixed and paraffin-embedded tissues were sectioned at a thickness of 4 μm and stained using an automated immunostainer (Discovery XT; Ventana Medical Systems, Tucson, AZ, USA). The slides were dried at 60°C for 1 hour and deparaffinized using EZ Prep (Ventana Medical Systems) at 75°C for 8 minutes. Cell conditioning was performed using CC1 solution (Ventana Medical Systems) at 100°C for 48 minutes. MMP-9 antibody (rabbit polyclonal antibody, 1:50 dilution; Diagnostic Biosystems, Pleasanton, CA, USA) was applied to the slides and incubated at 37°C for 32 minutes. Signals were detected using a DAB Map Detection Kit (Ventana Medical Systems). Counterstaining was performed with hematoxylin (Ventana Medical Systems) for 4 minutes at room temperature. We performed immunohistochemistry without the primary antibody as negative control.

### Evaluation of tumor MMP-9 expression

**Figure 1:**
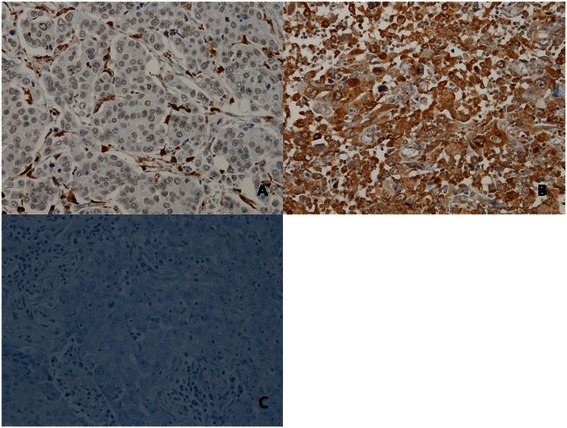
**Immunohistochemical analyses of NSCLC representing different expression levels for tumor MMP-9. (A)** No tumor MMP-9 expression; **(B)** tumor MMP-9 expression; **(C)** negative control.

IHC staining of tumor sections was reviewed and scored independently by 2 observers who were blinded to the clinical data. The staining intensity was classified as absent (score of 0), weak (score of 1), moderate (score of 2),or strong (score of 3). The extent of staining, defined as the percentage of positively stained cancer cells, was evaluated using a continuous scale (range, 0-100 %). The final IHC score for tumor MMP-9 expression was obtained by multiplying the staining intensity and the extent of staining. For statistical analysis, expression levels were classified according to the median IHC score (IHC score = 10) as no tumor MMP-9 expression (IHC score < 10) or tumor MMP-9 expression (IHC score ≥ 10) (Figure 1).

### Statistical analysis

Differences in MMP-9 expression and clinicopathologic variables were analyzed using the χ^2^ test. Age was dichotomized at the median value. Disease-free survival (DFS) was defined as the time from surgery to lung cancer recurrence, and overall survival (OS) was defined as the time between surgery and death from any cause. Relapse was defined as diagnosis of distant metastasis or local recurrence. Postoperative survival was analyzed using the Kaplan-Meier method and compared using the log-rank test. Multivariate analysis was performed using the Cox proportional hazards regression model with a forward selection procedure to study the effects of different variables on recurrence and survival. A p value less than 0.05 was considered statistically significant. All statistical manipulations were performed using the SPSS software program (SPSS Inc., Chicago, IL, USA).

## Results

### Patient population

A total of 417 patients were included in the study. The study included 279 men and 138 women, with a median age of 61 years (range, 30-81 years). The median follow-up period was 56.9 months (range, 3-168 months), and no patients were lost to follow-up. Relapse was observed in 114 patients, and in total, 150 patients died during the observation period. The patients’ characteristics are shown in Table 1.

**Table 1:** **Patient characteristics**

	Tumor MMP-9		P
	No Expression*N*(%)	Expression*N*(%)	
Age			0.820
Below median	137 (53.5)	88 (54.7)	
Above median	119 (46.5)	73 (45.3)	
Sex			0.099
Male	179 (69.9)	100 (62.1)	
Female	77 (30.1)	61 (37.9)	
Histology			0.001
Squamous cell	130 (50.8)	45 (28.0)	
Adenocarcinoma	103 (40.2)	99 (61.5)	
Large cell	11 (4.3)	9 (5.5)	
Others	12 (4.7)	8 (5.0)	
Stage			0.001
I	158 (61.7)	77 (47.8)	
II	70 (27.4)	51 (31.7)	
IIIA	28 (10.9)	33 (20.5)	
T stage			0.068
T1	75 (29.3)	33 (20.5)	
T2	147 (57.4)	105 (65.2)	
T3	32 (12.5)	18 (11.2)	
T4	2 (0.8)	5 (3.1)	
N stage			0.002
N0	204 (79.7)	108 (67.1)	
N1	35 (13.7)	30 (18.6)	
N2	17 (6.6)	23 (14.3)	
Lymphovascular invasion			0.549
No	224 (87.5)	144 (89.4)	
Yes	32 (12.5)	17 (10.6)	
Postoperative Treatment			0.000
Chemotherapy	73 (28.5)	71 (44.1)	
Radiation therapy	7 (2.7)	2 (1.2)	
Combination therapy	19 (7.4)	20 (12.4)	
No treatment	157 (61.3)	68 (42.2)	

MMP-9: matrix metalloproteinase-9; Combination therapy: Chemotherapy + Radiation therapy.

### Evaluation of MMP-9 expression

Of the 417 patients, 256 (61.4%) did not show tumor MMP-9 expression and 161 (38.6%) did show tumor MMP-9 expression. There was no significant difference in age, sex, and lymphovascular invasion (LVI) between these 2 groups. The group with tumor MMP-9 expression had a higher proportion of patients with adenocarcinoma histology (40.2% *vs.* 61.5%; *p* = 0.001) and more advanced stage (*p* = 0.001). Adjuvant therapy was administered more often in tumor MMP-9 expression group (38.7% *vs.* 57.8%; *p* = 0.000), but there was no difference in the frequency of adjuvant therapy for patients with stage II or IIIA (72.4% *vs.* 71.4%; *p* = 0.879). Occurrence of relapse (21.9% *vs.* 36.0%; *p* = 0.002) and death (34.8% *vs.* 37.9%; *p* = 0.518) was higher among patients with tumor MMP-9 expression.

### Analysis of DFS and OS

**Figure 2:**
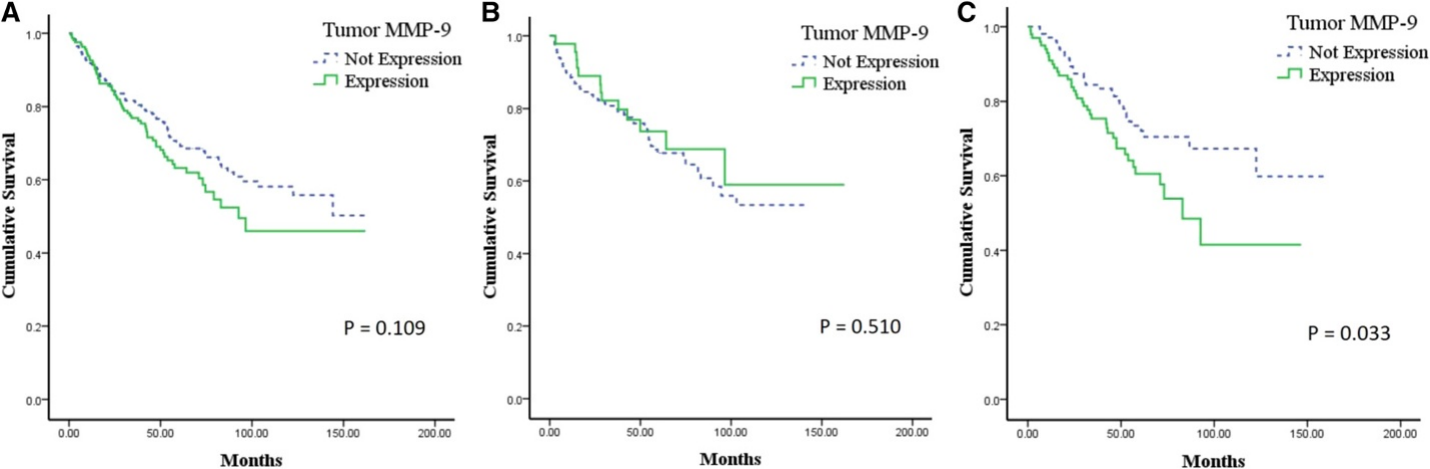
**Kaplan-Meier survival curves of the relationship between tumor MMP-9 expression and disease-free survival according to tumor histology. (A)** Overall tumor histology; **(B)** Squamous cell carcinoma; **(C)** Adenocarcinoma.

**Figure 3:**
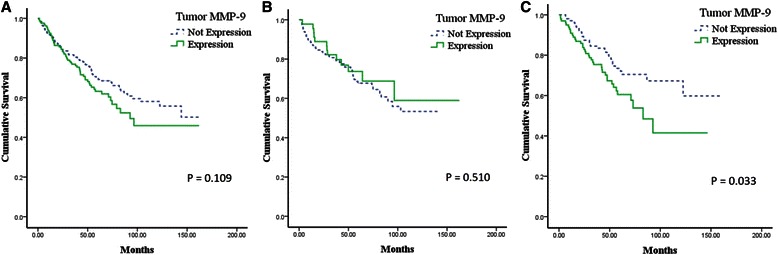
**Kaplan-Meier survival curves of the relationship between tumor MMP-9 expression and overall survival according to tumor histology. (A)** Overall tumor histology; **(B)** Squamous cell carcinoma; **(C)** Adenocarcinoma.

Univariate analysis revealed a relationship between tumor MMP-9 expression and DFS in patients with operable NSCLC. Patients with tumor MMP-9 expression had a shorter DFS than those without tumor MMP-9 expression (*p* = 0.01; Figure 2A). However, there was no significant correlation between tumor MMP-9 expression and OS (*p* = 0.109; Figure 3A).

Cox regression analysis was performed to evaluate the correlation between tumor MMP-9 expression and clinical outcomes. Tumor MMP-9 expression, age, LVI, tumor histology, and tumor stage were tested as independent possible prognostic variables. The results demonstrated that tumor MMP-9 expression was not a significant independent prognostic predictor for DFS (*p* = 0.142), whereas LVI, stage, and tumor histology were significant independent prognostic variables (Table 2). Similarly, MMP-9 expression was not an independent predictor for OS (*p* = 0.807); LVI and tumor stage were the only significant prognostic indicators for OS (Table 3).

**Table 2:** **Multivariate analysis of disease-free survival**

Prognostic factor	β	P	Relative risk	95% confidence interval
Overall histology				
Tumor MMP-9 expression	0.286	0.142	1.332	0.908–1.952
Lymphovascular invasion	0.586	0.028	1.797	1.065–3.032
Pathologic stage (I vs. II)	1.264	0.000	3.538	2.259–5.541
Pathologic stage (I vs. IIIA)	1.404	0.000	4.070	2.450–6.760
Tumor histology (squamous cell carcinoma vs. adeno)	0.685	0.002	1.985	1.299–3.033
Squamous cell carcinoma				
Tumor MMP-9 expression	−0.411	0.309	0.663	0.301–1.463
Adenocarcinoma				
Tumor MMP-9 expression	0.580	0.035	1.787	1.041–3.067

MMP-9: matrix metalloproteinase-9; adeno: adenocarcinoma.

**Table 3:** **Multivariate analysis of overall survival**

Prognostic factor	β	P	Relative risk	95% confidence interval
Overall histology				
Tumor MMP-9 expression	0.043	0.807	1.044	0.741–1.469
Lymphovascular invasion	0.661	0.005	1.937	1.219–3.078
Pathologic stage (I vs. II)	0.904	0.000	2.469	1.686–3.616
Pathologic stage (I vs. IIIA)	1.123	0.000	3.073	1.938–4.875
Squamous cell carcinoma				
Tumor MMP-9 expression	−0.419	0.196	0.658	0.349–1.241
Adenocarcinoma				
Tumor MMP-9 expression	0.295	0.259	1.343	0.805–2.242

MMP-9: matrix metalloproteinase-9.

### Analysis of DFS and OS by tumor histology

Clinicopathologic findings according to tumor histology are shown in Table 4. In patients with squamous cell carcinoma, tumor MMP-9 expression was not significantly associated with DFS (*p* = 0.639: Figure 2B) or OS (*p* = 0.510; Figure 3B). The results of the Cox regression analysis showed that tumor MMP-9 expression was not an independent prognostic indicator for DFS (Table 2) or OS (Table 3).

**Table 4:** **Patient characteristics by tumor histology**

	Tumor MMP-9					
	Squamous cell carcinoma			Adenocarcinoma		
	No Expression	Expression	P	No Expression	Expression	P
	*N*(%)	*N*(%)		*N*(%)	*N*(%)	
Age			0.464			0.160
Below median	64 (49.2)	25 (55.6)		57 (55.3)	45 (45.5)	
Above median	66 (50.8)	20 (44.4)		46 (44.7)	54 (54.5)	
Sex			0.021			0.166
Male	121 (93.1)	36 (80.8)		41 (39.8)	49 (49.5)	
Female	9 (6.9)	9 (20.2)		62 (60.2)	50 (50.5)	
Stage			0.021			0.006
I	65 (50.0)	15 (33.4)		77 (74.7)	55 (55.6)	
II	49 (37.7)	19 (42.2)		15 (14.6)	24 (24.2)	
IIIA	16 (12.3)	11 (24.4)		11 (10.7)	20 (20.2)	
T stage			0.042			0.019
T1	29 (22.3)	4 (8.9)		42 (40.8)	24 (24.2)	
T2	75 (57.7)	29 (64.5)		55 (53.3)	67 (67.7)	
T3	25 (19.2)	10 (22.2)		5 (4.9)	5 (5.1)	
T4	1 (0.8)	2 (4.4)		1 (1.0)	3 (3.0)	
N stage			0.183			0.008
N0	93 (71.6)	28 (62.2)		90 (87.4)	68 (68.7)	
N1	31 (23.8)	13 (28.9)		3 (2.9)	14 (14.1)	
N2	6 (4.6)	4 (8.9)		10 (9.7)	17 (17.2)	
Lymphovascular invasion			0.832			0.740
No	111 (85.4)	39 (86.7)		94 (91.3)	89 (89.9)	
Yes	19 (14.6)	6 (13.3)		9 (8.7)	10 (10.1)	
Postoperative Treatment			0.299			0.001
Chemotherapy	41 (31.5)	18 (40.0)		24 (22.3)	42 (42.4)	
Radiation	7 (5.4)	1 (2.2)		0	1 (1.0)	
Combination	10 (7.7)	6 (13.3)		7 (6.8)	13 (13.1)	
No treatment	72 (55.4)	20 (44.4)		72 (69.9)	43 (43.4)	

MMP-9: matrix metalloproteinase-9; Combination: Chemotherapy + Radiation therapy.

However, among patients with adenocarcinoma, there was a significant negative correlation between tumor MMP-9 expression and both DFS (*p* = 0.003; Figure 2C) and OS (*p* = 0.033; Figure 3C). Cox regression analysis was used to define clinical markers with independent predictive value with respect to DFS and OS. Tumor MMP-9 positivity was an independent prognostic factor for DFS (*p* = 0.035; Table 2); however, OS was not associated with MMP-9 expression (*p* = 0.259; Table 3).

## Discussion

MMPs are a group of zinc-dependent endopeptidases that have been implicated in the degradation of extracellular matrix. The role of MMPs in tumor growth, metastasis, and angiogenesis has been widely investigated [18]. MMPs are divided into 4 subclasses according to their substrate specificity: collagenases, gelatinases, stromelysins, and elastases [19]. Among these, MMP-9 (gelatinase-B), a crucial factor in angiogenesis, plays a critical role in the progression of a variety of tumor types.

Expression levels of MMP-9 in serum and tissue are significantly higher in patients with pancreatic ductal adenocarcinoma than in those with pancreatitis [20], and tumor MMP-9 expression is significantly elevated in patients with breast cancer [21]. MMP-9 has been studied as a prognostic factor for colorectal cancer in T3–T4 node-negative patients; enhanced tumor MMP-9 expression was found to be an independent marker of poor prognosis [22]. However, in ovarian cancer, tumor MMP-9 expression is associated with longer survival, whereas stromal MMP-9 expression is associated with shorter survival [23].

Many recent reports of tumor MMP-9 expression in patients with operable NSCLC have suggested that tumor MMP-9 expression is a predictor of poor prognosis [12,14,15]. However, the prognostic impact of IHC detection of tumor MMP-9 expression in operable NSCLC is controversial [16-19]. We therefore performed this study to assess the prognostic impact of tumor MMP-9 expression, as determined by IHC staining, in patients with operable NSCLC.

There are certain limitations to studies of tumor MMP-9 expression that may limit the interpretation of the results. First, there were differences among previous studies in the definition of tumor MMP-9 positivity and the appropriate cut-off value. Most of these studies applied a scoring system that was based on the extent and intensity of staining for tumor MMP-9 expression and showed that overexpression of tumor MMP-9 was associated with a poor prognosis [12,14,15]. Another study used the median value of staining for tumor MMP-9 to define tumor MMP-9 positivity [24]. However, there is no common cut-off value for defining positive tumor MMP-9 expression in NSCLC. In this study, an IHC score was used to determine tumor expression levels of MMP-9, and the median IHC score was used as the cut-off value [25]. An IHC staining score of 10 (range, 0-300) was used to divide patients into 2 groups, according to the presence or absence of tumor MMP-9 expression. In other words, an IHC score of less than 10 was defined as an absence of tumor MMP-9 expression. The important point of our study was that tumor MMP-9 positivity was not determined by a scoring system but by the presence or absence of tumor MMP-9 expression. Despite efforts to standardize this process, a widely accepted scoring system has not yet been established. Thus, our dichotomous distinction for tumor MMP-9 expression will reduce the impact of subjective judgment when determining tumor MMP-9 positivity. In our study, 38.6% of patients had tumor MMP-9 expression. However, if a cut-off value of 20% was applied, tumor MMP-9 positivity would be reduced to 27.3%. Our result of 38.6% seems reasonable because it is consistent with the percentage of patients showing tumor MMP-9 positivity in previous studies (range, 29.4-68.9 %) [12,14]. The patients of tumor MMP-9 expression had more advanced stage (*p* = 0.001; Table 1), therefore, they received more postoperative treatment (*p* = 0.000; Table 1). However, there was no difference in adjuvant therapy for patients with stage II or IIIA (72.4% vs. 71.4%; *p* = 0.879) and there was no statistical difference in adjuvant therapy for histological type.

Second, it is important to interpret data according to tumor histology. In previous studies, all types of tumor histology were included in the analysis of tumor MMP-9 expression, and the value of the prognostic factors identified in these studies is controversial [16-19]. Some reports have indicated that tumor MMP-9 expression is a prognostic factor for adenocarcinoma of the lung [11,24], and we analyzed that a higher proportion of the adenocarcinoma patients had positive tumor MMP-9 expression than squamous carcinoma in this study (28.0% vs. 61.5%; *p* = 0.001). Thus, histological distinction (squamous cell carcinoma *vs.* adenocarcinoma) seems to be necessary in the analysis of tumor MMP-9 expression. In the present study, the overall cohort included patients with all histological types, including squamous cell carcinoma, adenocarcinoma, and large cell carcinoma. When considering the total population, tumor MMP-9 expression was associated with an increased risk of relapse (*p* = 0.01; Figure 2A) but was not predictive of OS (*p* = 0.109; Figure 3A). Furthermore, when including all tumor types, tumor MMP-9 expression was not an independent prognostic factor for the additional outcomes tested. After stratifying by tumor histology (squamous cell carcinoma *vs.* adenocarcinoma), tumor MMP-9 expression was associated with a poor prognosis for relapse (*p* = 0.003; Figure 2C) and OS (*p* = 0.033; Figure 3C) and was an independent prognostic factor for relapse (*p* = 0.035; Table 2) of adenocarcinoma of the lung. Moreover, previous studies included only limited analyses of tumor MMP-9 expression in early-stage adenocarcinoma, whereas this study evaluated stage I–IIIA operable adenocarcinoma of the lung [14,24].

Finally, it is difficult to analyze survival outcome according to tumor MMP-9 expression. Survival outcomes include both relapse and survival. MMP-9 is known to be a key factor in the degradation of the extracellular matrix and angiogenesis, processes related to tumor metastasis. Therefore, it is reasonable to evaluate tumor MMP-9 expression with respect to relapse, which includes both recurrence and metastasis, rather than with respect to survival. Most previous studies have defined the association of tumor MMP-9 expression in NSCLC with a poor survival prognosis [12,14,15], and only a few studies have shown the value of tumor MMP-9 expression for predicting relapse [18]. Our study demonstrated that tumor MMP-9 expression was a significant and independent prognostic factor for the relapse of lung adenocarcinoma.

## Conclusions

Tumor MMP-9 expression correlated with relapse in operable NSCLC patients; however, we were not able to demonstrate the clinical significance of tumor MMP-9 expression as a prognostic marker for relapse and survival. However, subgroup analyses of tumor histology suggested that tumor MMP-9 expression was associated with decreased DFS and OS in patients with adenocarcinoma but not in those with squamous cell carcinoma. Moreover, in this study, Cox regression analysis revealed that tumor MMP-9 expression was an independent poor prognostic factor for the relapse of lung adenocarcinoma. Thus, more studies will be needed to confirm this, and furthermore, IHC staining to distinguish tumor MMP-9 expression may be useful to predict clinical outcomes after surgical resection of lung adenocarcinoma.

## Consent

Written informed consent was obtained from the patient for the publication of this report and any accompanying images.

## Abbreviations

MMP-9: Matrix metalloproteinase-9
NSCLC: Non-small cell lung cancer
DFS: Disease-free survival
OS: Overall survival
